# Effect of High Hydrostatic Pressure in the Storage of Spanish-Style Table Olive Fermented with Olive Leaf Extract and *Saccharomyces cerevisiae*

**DOI:** 10.3390/molecules27062028

**Published:** 2022-03-21

**Authors:** Daniel Martín-Vertedor, Thais Schaide, Emanuele Boselli, Manuel Martínez, Jesús García-Parra, Francisco Pérez-Nevado

**Affiliations:** 1 Technological Institute of Food and Agriculture (CICYTEX-INTAEX), Junta of Extremadura, Avda. Adolfo Suárez s/n, 06007 Badajoz, Spain; jjgparra@gmail.com; 2 Research Institute of Agricultural Resources (INURA), Avda. de la Investigación s/n, Campus Universitario, 06006 Badajoz, Spain; tschaide@alumnos.unex.es (T.S.); mmcano@unex.es (M.M.); fpen@unex.es (F.P.-N.); 3 Área de Nutrición y Bromatología, Departamento de Producción Animal y Ciencia de los Alimentos, Escuela de Ingenierías Agrarias, Universidad de Extremadura, 06007 Badajoz, Spain; 4 Faculty of Science and Technology, Free University of Bozen-Bolzano, Piazza Università 1, 39100 Bolzano, Italy; emanuele.boselli@unibz.it; 5 Área de Producción Vegetal, Departamento de Ingeniería del Medio Agronómico y Forestal, Escuela de Ingenierías Agrarias, Universidad de Extremadura, 06007 Badajoz, Spain

**Keywords:** yeast, chemical parameters, phenols, HHP, sensory attributes, table olives

## Abstract

Olives treated according to the Spanish-style are firstly treated with caustic soda and then fermented in brine to reduce phenols. Next, olives are packed and subjected to pasteurization. The effect of different high hydrostatic pressure treatments (400 MPa, 4 and 6 min) was evaluated in Spanish-style table olives fermented with olive leaf extract (OLE) and *S. cerevisiae* compared with thermal pasteurization (P) at 80 °C for 15 min. HHP and P led to a significant reduction in yeast and aerobic mesophiles after the conservation treatment and during storage (300 days). The physical–chemical properties changed slightly during storage, except for olive hardness; olives treated with HHP presented a higher hardness than pasteurized ones. The CIELAB parameter L* decreased until day 300 in most of the treatments, as well as phenols. The HHP treatment led to significantly higher contents of phenolics (even during storage) than olives submitted to P. Some sensory attributes (colour, aspect, hardness, and overall evaluation) decreased during storage. P treatment caused a decrease in appearance, aroma, hardness, and overall evaluation compared to olives treated with HHP. Thus, the application of HHP in table olives to increase the shelf-life can be considered a valid alternative to P.

## 1. Introduction

Olive cultivation is mainly concentrated in Mediterranean countries. Spain is the country with the largest amount of land dedicated to this crop, around 2.69 million ha [1] which corresponds to more than 180 million olive trees [2]. In Spain, the ‘Hojiblanca’ variety is the main table olive treated according to the Spanish-style, followed by ‘Carrasqueña’ or Manzanilla de Sevilla’ and ‘Manzanilla Cacereña’ [1]. These varieties are important in the regions of Andalusia and Extremadura (southern and southwest of Spain, respectively). Among fermented foods, table olives are considered the most popular vegetable food specialty in the Mediterranean region, being widely consumed for their organoleptic characteristics and for the contribution of bioactive compounds present in the olives (monounsaturated fatty acids, polyphenols, vitamins, and minerals) [3].

In the table olive industry, the Spanish-style is the most common process, where olives are treated with caustic soda to remove bitterness and then fermented for several months in a salt solution. This is one of the critical points during the treatment process because there is a complex microbial biodiversity that often affects their organoleptic characteristics, giving the product a distinctive flavour [4]. De Angelis et al. [5] showed the beneficial role of yeasts in table olive fermentation, which contribute to improve the flavour of the fermented product. For this reason, there is currently a growing concern in selecting microorganisms that have optimal technological properties and beneficial probiotic characteristics [6].

The olive industry generates a large amount of by-products annually that are mostly not used [7]. Among these by-products, olive leaves stand out, accounting for 10% of the total weight of the harvest [8]. Olive leaves are an economic raw material that can be used as a suitable source of high added value products [8,9,10]. Thus, several researchers have developed different olive leaves extracts (OLE) that are used as additives as a source of naturally derived antioxidants due to the high content of phenolic compounds [11,12,13,14,15].

Currently, emerging food preservation technologies are increasingly present in the food industry, due to the increasing demand of food quality and safety [16]. High hydrostatic pressure (HHP) gains relevance over traditional conservation methods that use heat treatments for microbial inactivation. The increase in temperature causes chemical changes in treated foods affecting physical, sensory, and nutritional properties [17]. However, the use of HHP at a refrigerated or room temperature allows the inactivation of spoilage and/or pathogenic microorganisms, but avoiding the negative consequences of conventional thermal treatments [18]. There are few studies reporting high hydrostatic pressure in table olive preservation; to achieve pasteurization, the pressure ranges from 400 to 600 MPa for 5–10 min [18,19,20,21]. In addition, this technique could ensure the preservation of bioactive compounds in table olive [22].

The purpose of this study was to monitor the microbiological and chemical characteristics of Spanish-style table olive fermented with OLE and *Saccharomyces cerevisiae* yeast by evaluating their stability during the storage after applying different high hydrostatic pressure treatments.

## 2. Results and Discussion

### 2.1. Evolution of the Microbiota during Storage

**Figure 1:**
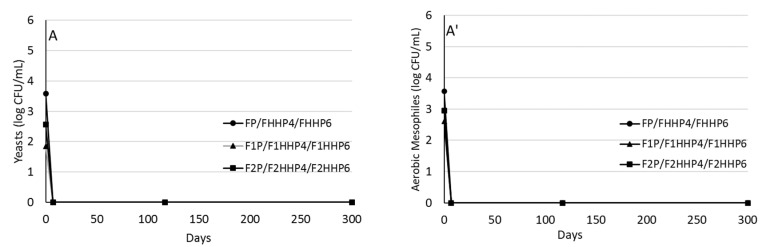

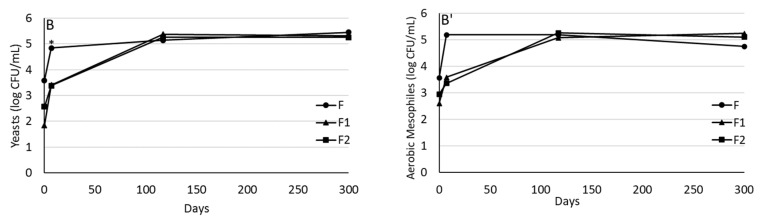
Evolution of yeasts counts and aerobic mesophilic microorganisms in pasteurized (FP, F1P, F2P), HHP 400 MPa 4 min (FHHP4, F1HHP4, F2HHP4) and HHP 400 Mpa 6 min (FHHP6, F1HHP, F2HHP6) treated table olives (**A**,**A’**) and untreated table olives (control) (**B**,**B’**). *: statistically significant differences (Tukey’s Test, *p* < 0.05) among conservation treatments at the same storage time.

Table olives submitted to high hydrostatic pressure (HHP) and thermal pasteurization (P) showed a significant reduction in yeast and aerobic mesophiles (Figure 1A). No microorganisms were detected in olives treated with HHP and P during the storage period of the product (300 d). However, in untreated table olives (F), an increase in aerobic mesophiles and yeast counts was observed from the first week of the storage period (Figure 1B). In olives fermented with olive leaf extracts (OLE) (F1 and F2), microbial development was slower. From day 120 of the storage period, all the treatments presented a similar concentration of microorganisms, approximately 5 CFU mL^−1^.

Our results show that HHP caused the decrease in microbial counts, which, based on the scientific literature, could be due to the fact that HHP provoke the induction of sublethal damage in the microorganism cell wall and membrane causing their death [23]. The low pH of Spanish-style olives (pH < 4) may also contribute to microbial control during the storage period [24,25].

It must be noted that the reduction of yeasts and aerobic mesophiles was similar using both time intervals for HHP (400 MPa for 4 and 6 min). No lactic acid bacteria, enterobacteria, coliforms, *Bacillus*, and *Pseudomonas* were detected either in the fermented olives or during storage (data not shown). Thus, a treatment of 400 MPa for 4 min would be sufficient to control the growth of microorganisms in Spanish-style table olives, ensuring microbial quality for at least 10 months; these processing conditions ensure food safety and suitability of the product for consumption according to the recommendations of Codex Alimentarius [26].

The mechanism of inactivation of vegetative microorganisms by high-pressure conditions is a combination of different reactions, such as the breaking of non-covalent bonds, increased permeability in the cell membrane and a decrease in the intracellular pH due to the modification of ion outflow by membrane disruption. At low levels of pressure, the morphological damage to the cell membrane is often reversible; whereas at higher levels of inactivation of microorganisms, irreversible damage occurs [27]. Several authors indicated more effective microbial inactivation by applying treatments at higher pressures (600 vs. 450 MPa) and longer treatment times (10 vs. 5 min) [28,29,30]. Similar results have been obtained in our study with the difference that the fermentation with OLE provoked higher olive quality due to the increase in the phenolic content. Abriouel et al. [18] indicated the effect of the storage of ‘Aloreña’ olives variety submitted to HHP observing that treatment at 300 to 400 MPa for 5 min caused the inactivation of the yeast populations during three months of storage. Sánchez et al. [30] and Delgado-Adámez et al. [28] found that HHP was effective to control the growth of mesophilic aerobes, yeasts, and other microorganisms in table olive products. These researchers have indicated that the product treated with HPP at 600 MPa for 5 min was microbiologically stable for 18 months. Researchers indicated that pressure values between 200–600 MPa at room temperature are usually sufficient to inactivate most vegetative cells, including moulds and yeasts [31].

### 2.2. Evolution of Physical-Chemical Parameters during the Storage Period

The results obtained from the regular monitoring of physical–chemical parameters (pH, total chlorides, and free acidity) for 300 days of storage time are shown in Table 1. The initial pH of samples was around 3.5, while free acidity was 0.3% lactic acid and chlorides were close to 4.0 g 100 mL^−1^ NaCl. In general terms, the slight changes found in pH and free acidity at the beginning of storage cannot be considered representative as they correspond to centesimal differences in these values. However, when samples were stored for 300 days, physical–chemical values were not significantly different in table olives produced with and without inoculation with *Saccharomyces cerevisiae* and/or the addition of OLE. However, in the different treatments (P, HHP4, and HHP6), the pH decreased and free acidity and chloride concentration increased slightly during storage. Therefore, HHP treatment showed similar effects to pasteurization on the reported parameters. These table olives might be suitable for introduction in the market, in fact IOC [32] indicated that the maximum pH of table olives must be 4.0.

Pradas et al. [20] found a reversible effect of HHP on pH and acidity due to a rebalancing of acidic compounds between olives and brine. However, several studies found an increase in pH in the brine during storage while lactic acid decreased [20,33]. Instead, García-Parra et al. [29] found a decrease in pH during storage in fermented food products probably due to the lactic fermentation of sugars leading to acidification. In addition, Rodríguez-Gómez et al. [33] showed a slight increase in chlorides which the authors claim as increased product stability.

**Figure 2:**
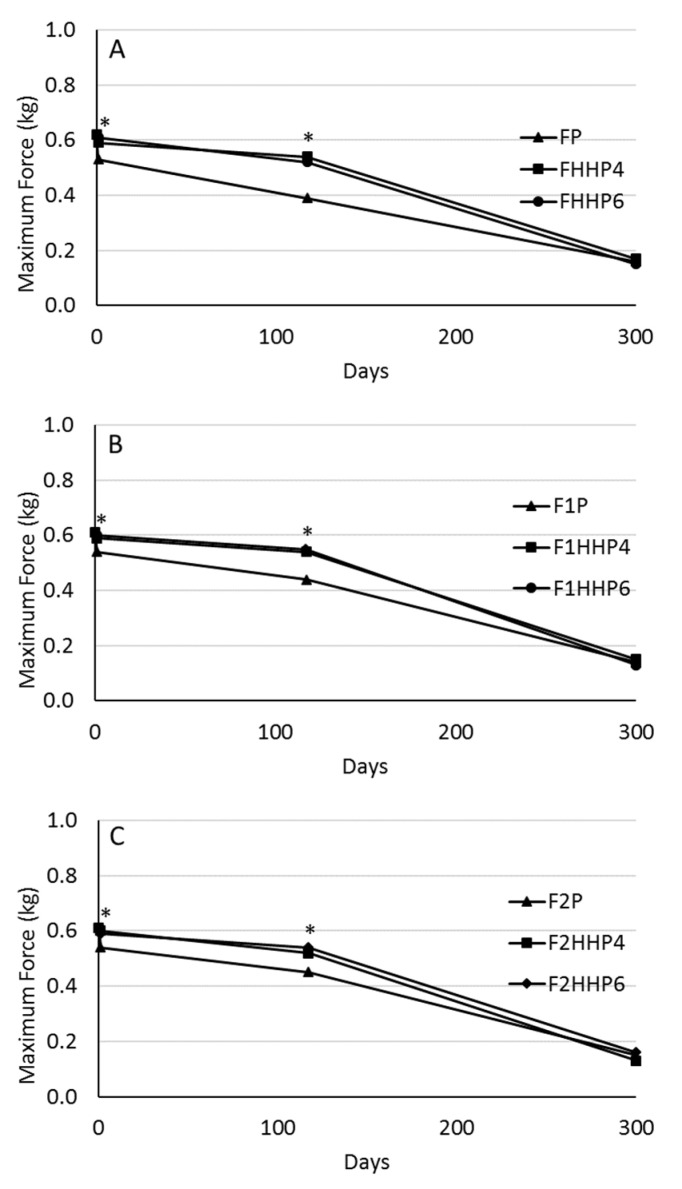
Hardness evolution of table olives during the storage process. (**A**): control olives, without inoculation or OLE addition (FP, pasteurized; FHHP4 and FHHP6, treated with HHP); (**B**): olives with OLE and non-inoculated (F1P, pasteurized; F1HHP4 and F1HHP6, treated with HHP); (**C**): olives inoculated with *S. cerevisiae* strain and added with OLE (F2P, pasteurized; F2HHP4 and F2HHP6, treated with HHP). *: indicates statistically significant differences (Tukey’s Test, *p* < 0.05) among conservation treatments for the same storage time.

The trend in hardness of the treated olives during storage is shown in Figure 2. The maximum force was also monitored to determine the effect of P and HHP on the texture of the olives. The olives showed a decrease in hardness during storage. In general, a significant decrease in the maximum force was observed between day 117 and 300. Olives treated thermally (P) and olives treated with HHP (HHP4 and HHP6) showed significant differences from day 0 to day 117; however, HHP4 and HHP6 olives did not show differences during the storage period. There was a significant loss of hardness after 117 days of storage; therefore, the texture of the olives was too soft to recommend consumption. This result is interesting because olives treated with HHP presented a good hardness during a 117-day storage period. Similar results were found by Pradas et al. [20], who observed that also table olives of a different variety (Cornezuelo) which were subjected to HHP showed a decrease in hardness during storage. In addition, Güngör et al. [34] using different preservation methods, such as pasteurization, addition of sorbate salts, and ozone treatment, found hardness losses along the process.

Colour is one of the most important attributes of sensory properties to consumers. Casado et al. [35] indicated that consumers prioritize olive fruits with green tones, as they tend to correlate it with “greater freshness” or a “more natural or less processed product”. Analysing the different colour parameters (CIELAB colour space) of olives during the storage period, it can be observed that the highest values for the brightness parameter (L*) were found in olives at day 0. From this date, a decrease was observed in most of the treatments studied until day 300 (Table 2). In general, heat-treated (P) and high pressure treated olives showed a different pattern for the L* parameter. Comparing the treated olives each day of the storage time, higher values were found in high pressure treated olives.

On the other hand, a general increase in the values of the a* parameter (varying from green to red) was observed during the storage period. Initially the olives showed a more greenish colour, which decreased during storage. Some differences were observed according to the treatment and fermentation process applied. In general, green colour degradation was observed in control olives (F) and in olives with OLE but not inoculated (F1), during storage. However, in olives fermented with OLE and inoculated with yeasts (F2), some differences were found between treatments; pasteurized olives did not show a loss of green colour, but HHP treated olives lost this colour through the storage period. When comparing heat-treated olives with HHP treated olives, P olives showed a greener colour each day of the storage process. In addition, the determination of the b* parameter in the CIELAB space showed a decrease in the yellow colour of the olives during the storage. In the control (F) the smallest differences were observed during storage between heat-treated and FHHP4 olives; however, in the olives fermented with OLE (F1) and in the olives fermented with OLE and inoculated (F2), F2HHP4 and F2HHP6 treatments showed the smallest differences. Comparing by storage time, in general, pasteurized olives maintained the yellow colour better than those treated with HHP. At the end of the storage period (day 300), the highest values of the L* parameter were found in the control olives without conservation treatment (F), indicating that the olives were brighter than the other treatments. Additionally, more differences were found in the values of the a* parameter; the olives from F1 and F2 processes with pasteurization treatment (P) were the greenest ones. The F1 pasteurized and F with HHP treatment for 4 min were the most yellowish olives. The results obtained indicate that the addition of OLE would not be particularly effective in protecting the olives from losing the green colour. It should be noted that the olives were processed by adding OLE and inoculating a yeast strain at the beginning of fermentation; this may explain why a non-significant effect was observed during the storage period.

Casado et al. [35] also indicated that, due to heat treatments, olives evolve toward a browner hue, which is consistent with the results obtained in this study. Clydesdale and Francis [36] showed that the loss of green colour during heat treatment is due to the formation of pheophytin (gray-brown colour) from chlorophylls (bright green colour). On the other hand, the yellowish colour in the olives processed according to the Spanish-style is mainly due to the treatment with alkali. The colour changes toward a browner colour after their conservation is due to polymeric substances of phenolic nature and heavier than 10,000 Daltons which are formed by enzymatic degradation [37]. Due to the auto-oxidation of olives over time, loss of pigments occurs resulting in a darker colour [38].

### 2.3. Profile of Phenolic Compounds

The profile of phenolic compounds in table olives subjected to different storage treatments and stored for 10 months is shown in Table 3. The main phenols detected were hydroxytyrosol, tyrosol, oleuropein, procyanidin B1, vanillic acid, p-coumaric acid, and verbascoside. The content of phenols decreased during storage by about 40% in all the conservation treatment applied. Furthermore, the application of P treatment significantly decreased the content of most of the phenols after the application of the conservation treatment and after the storage period. Similar results were found by Fernández et al. [21] in four different varieties of table olives. However, the HHP treatment caused significantly higher contents of phenolic compounds, even during storage. Moreover, the concentrations of seven phenols decreased by applying pasteurization. The same results were found by Geraldi et al. [39], who observed a decrease in the phenolic concentration when HHP treatments were applied. Moreover, the addition of *Saccharomyces cerevisiae* and OLE during fermentation (F2) provoked a decrease in concentration of phenolic compounds compared to the addition of OLE alone (F1).

It should also be noted that olives fermented with OLE were those that had the highest content of phenolic compounds. In fact, olives fermented without starter and with OLE (F1) presented almost twice as much phenolics as F without added OLE. Furthermore, olives fermented with *Saccharomyces cerevisiae* and OLE (F2) had almost 40% less phenols. This may be due to the fact that yeasts metabolize part of these compounds during the fermentation process. Fernández et al. [21] showed that certain microorganisms were able to reduce phenols in table olives during the fermentation process. Similarly, Caponio et al. [14] studied table olives fermented with the inoculation of *L. plantarum* and found a significant increase in hydroxytyrosol and verbascoside in the olive pulp. The most abundant phenolic compounds in the olive pulp were hydroxytyrosol, verbascoside, luteolin and rutin.

It is necessary to take into account that hydroxytyrosol has been associated with oleuropein degradation [40] and the diffusion of phenols from olive fruit to brine. In this work, the inoculated yeasts could have promoted the hydrolysis of phenolics. However, the results in brine were very different. The results obtained by Lalas et al. [41] are comparable to our results in the olive pulp, showing an increase in oleuropein and hydroxytyrosol in table olives treated with an OLE. Oleuropein and hydroxytyrosol are associated with positive health effects and Martín-Vertedor et al. [12] indicated that OLE showed antioxidant, antimicrobial and antitumor in vitro activities after simulated digestion.

Different researchers have indicated that the processing of foods using HHP could minimize the degradation of functional molecules, thus ensuring satisfactory organoleptic, microbiological, physicochemical and antioxidant characteristics even after their storage [21,42]. Cao et al. [43] in their study of the application of HHP to strawberries indicated that the use of this technique increased the phenolic content of the fruit. These results agree with those of Huang et al. [44], who refer to the fact that HHP could lead to the inactivation of enzymes promoting (indirectly) an increase in phenols. Kaşikçi and Bağdatlioğlu [45] indicated that HHP is better than P treatment on retaining phenolic compounds. Compared to the cited previous literature, this study combined the evaluation of the quality changes of table olives treated with HHP during a storage period, together with the use of olive leaf extract (OLE), as a way to increase and/or maintain the content of phenolic compounds that are usually lost during processing.

### 2.4. Sensory Analysis

**Figure 3:**
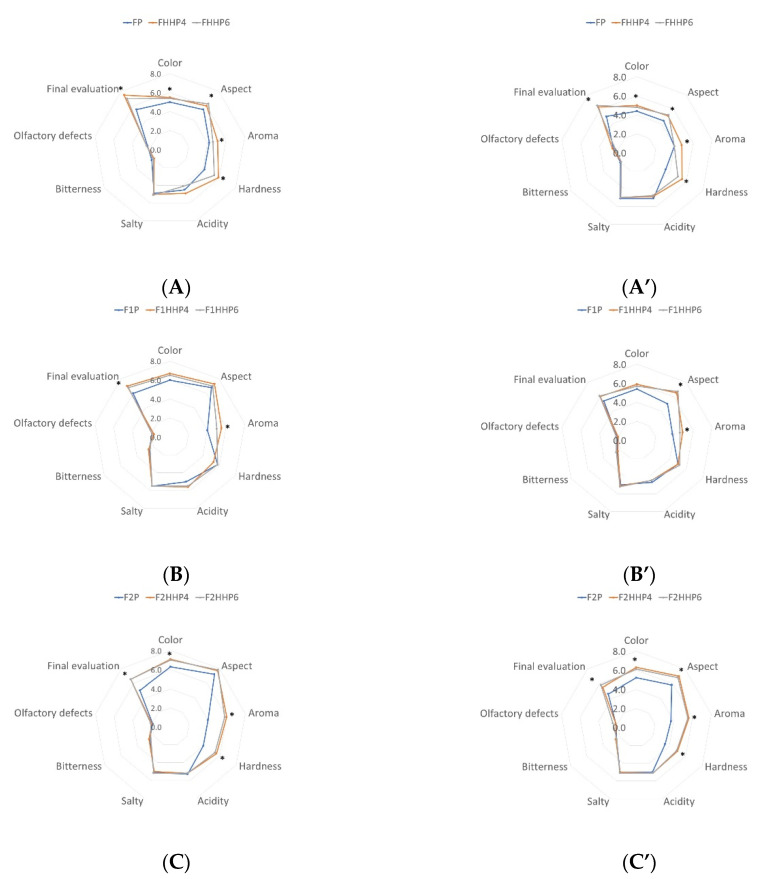
Spider plots of the sensory descriptors of fermented olives after the treatments (**A**–**C**) and after 117 days of storage (**A’**–**C’**). Control olives (F), without inoculation or OLE addition: FP, pasteurized; FHHP4 and FHHP6, treated with HHP. Olives with OLE and non-inoculated (F1): F1P, pasteurized; F1HHP4 and F1HHP6, treated with HHP. Olives inoculated with *S. cerevisiae* strain and added with OLE (F2): F2P, pasteurized; F2HHP4 and F2HHP6, treated with HHP. *: statistically significant differences (Tukey’s Test, *p* < 0.05) among conservation treatments at the same storage time.

The results obtained from the sensory analysis after subjecting the table olives to different storage treatments (day 0 and 117) are shown in Figure 3. Some significant differences were observed for the different treatments performed for each sensory attribute. The parameters that showed the most differences were “Color”, “Aspect”, “Aroma”, “Hardness”, and “Final evaluation”. The sensory evaluation was carried out until day 117 because by day 300 all the physical–chemical parameters showed values that made olives non-commercial. For the attribute “Color”, values ranged from 4.4 to 7.1. During storage, the values decreased slightly in the three types of fermentation even though all treatments presented fairly acceptable colour values (above 5 points). For “Aspect”, values ranged from 4.4 to 7.8. Values of this attribute decreased during storage. “Hardness” was rated between 3.5 and 5.9 showing that this attribute decreased during storage. The attributes “Aroma”, “Acidity”, “Salty”, “Bitterness”, and “Olfactory defects” did not show evident differences during the storage period. For the “Final evaluation” of the product, the values ranged from 4.6 to 7.5 showing a decreasing trend during storage.

The lowest values for the broad attributes evaluated were found for spontaneously fermented olives. It seems that olives fermented with OLE and/or *Saccharomyces cerevisiae* were those that had higher values in the sensory attributes. Yeast inoculation would ensure homogeneity throughout the fermentation process. This type of fermentation with yeasts through a controlled inoculation could cause a better sensory quality of olives. In this sense, other researchers [46] have indicated that yeast inoculation plays a role in the improvement of sensory characteristics of table olives.

In addition, it should be pointed out that fermentation with OLE caused good acceptance by the panel, an aspect that it is interesting because the extract was rich of phenols. The use of OLE during fermentation has been suggested by Schaide et al. [15] showing no bitterness and high acceptability of table olives obtained according to the Spanish-style.

The P treatment applied for olive preservation had a significant influence on some of the evaluated attributes. In fact, pasteurization caused a decrease in “Color”, “Aspect”, “Aroma”, “Hardness”, and “Final evaluation” compared to HHP-treated olives. Moreover, the application of different intensities during HHP treatment (400 MPa 4 vs. 6 min) did not cause significant differences in the sensory attributes. The work of Pradas et al. [20] on Spanish-style fermentation of ‘Cornezuelo’ variety showed that the application of 400 MPa for 5 min for HHP was related to the best acceptance up to 280 days of storage. Thus, the application of HHP appears to be an effective treatment used by table olive industries to market olives with satisfactory food safety and improved sensory profile also in the case of ‘Carrasqueña’ variety used in this study.

## 3. Materials and Methods

### 3.1. Samples

**Figure 4:**
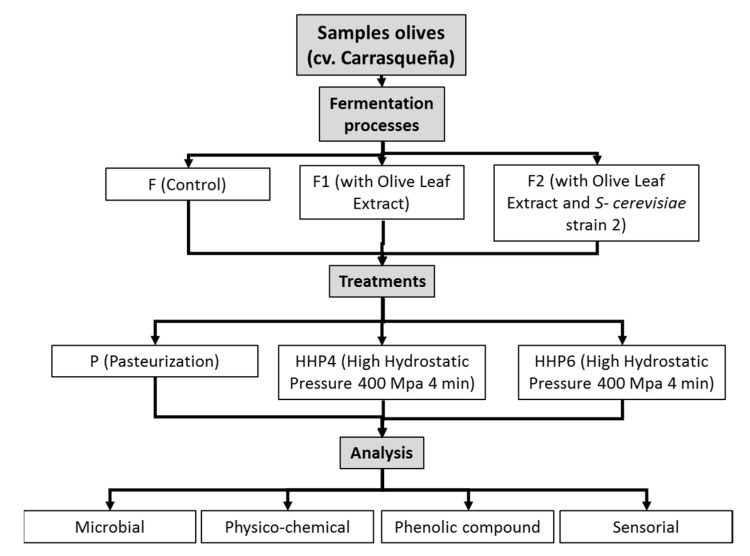
Diagram of the experimental design.

The olives (*Olea europaea* L.) and olive leaves of the ‘Carrasqueña’ variety were obtained in the research fields of the CICYTEX Center in Guadajira (Badajoz, Spain). A diagram of the overall experimental design is shown in Figure 4.

Three types of fermentations were performed in triplicate. The fermenters were filled with 30 kg of table olives with its corresponding brine solution. Olives were fermented for 121 days [15] using three types of processing:

- F: spontaneously fermented olives.
- F1: olives fermented spontaneously and with olive leaf extract (OLE).
- F2: olives fermented with a starter culture of a strain of *Saccharomyces cerevisiae* (6 log_10_ cfu/mL) and with OLE.

Once the fermentation process was completed, the olives were washed with water and immersed in the same volume of a governing liquid with a composition of 4.5% NaCl, 0.025% sorbic acid, 0.05% benzoic acid, 0.04% calcium chloride, and 0.8% lactic acid. Subsequently, the table olives were packaged and subjected to three different conservation treatments:

(I) Pasteurization heat treatment (P). A total of 125 g of table olives were packed in glass jars and treated in a 300 L horizontal rotary autoclave at 80 °C for 15 min. The temperature of the product inside the jars (200 mL) was automatically controlled.

(II) High hydrostatic pressures (HHP) 400 MPa for 4 min (HHP4). For this treatment, 250 g of table olives were packed in vacuum heat-sealed plastic polyethylene bags (9.3 mL O_2_/m^2^/24 h at 0 °C) and treated at 400 MPa for 4 min in an HHP commercial equipment with a capacity of 55 L (NC Hyperbaric Wave 6000/55 Burgos, Spain).

(III) High hydrostatic pressures (HHP) 400 MPa for 6 min (HHP6). This treatment was like the previous one, HHP4, with 250 g of table olives and treatment at 400 MPa; the only differences was that, in this case, the duration was 6 min.

The treated table olives were then stored in a conservation chamber at room temperature in darkness for 300 days. Throughout the fermentation, samples were taken on day 0, 117, and 300, and the corresponding analyses (microbiological, physical–chemical, phenolic profile, and sensorial) were carried out.

### 3.2. Microbiological Analysis

To determine the presence and concentration of different microorganisms, brine samples were taken aseptically, diluted with peptone water and plated in corresponding culture media. The microorganisms analysed were yeasts and moulds (YEPD Agar, 48 h at 30 °C), total aerobic mesophiles (PCA 48 h at 30 °C), *Pseudomonas* sp. (*Pseudomonas* Agar, 48 h at 30 °C), Lactic acid bacteria (MRS Agar, 48 h at 37 °C in anaerobic environment), *Bacillus cereus* (MYP Agar, 48 h at 37 °C), coliforms (VRBA Agar, 48 h at 37 °C), and *Enterobacteriaceae* (VRBG Agar, 48 h at 30 °C). All analysis were performed in triplicate.

### 3.3. Physicochemical Analysis

pH readings were taken during fermentation with a Basic 20 pH meter (Crison Instruments, Barcelona, Spain). Titratable acidity was determined with titration with sodium hydroxide (0.1 N) being expressed in grams of lactic acid 100 mL^−1^ of brine [47]. Total chlorides were determined by titration with silver nitrate according to Mohr’s method using potassium dichromate as indicator, the results being expressed as g 100 g^−1^ and performed in triplicate.

Olive colour analysis was performed periodically using a Minolta Chroma Meter CR-300 portable colorimeter (Minolta, Osaka, Japan) to determine the CIELAB coordinate space (L*: lightness; a*: green to red; b*: yellow to blue). Readings were taken for a total of 30 olives.

Texture analysis carried out on the different treatments during fermentation were performed using a TA.TX2 texturometer (Stable Microsystems, Surrey, UK), whose loading capacity is 30 kg. The needle used to make the probe had a capacity of 4 mm penetration. To evaluate the texture during fermentation, the parameter used was hardness. In this measure was expressed as maximum force (kg). The data for each measurement were 30 olives.

### 3.4. Determination of the Phenolic Profile

The profile of phenolic compounds was determined in fresh olives pulp during the storage period. Extraction of phenolic compounds was performed using the methodology described by Cabrera-Bañegil et al. [48]. Identification and quantification were carried out with HPLC model Agilent 1100 (Agilent Technologies, Palo Alto, CA, USA) controlled by ChemStation for LC 3D with system Rev. B.03.02 system.

### 3.5. Sensory Analysis

Sensory analysis was performed by a panel of eight trained tasters according to the tasting sheets proposed by González et al. [49] following the standardized norm of the International Olive Council [32]. The sensory properties of the brine and olive fruits, including colour, aspect, hardness, acidity, salty taste, bitter taste, aromas, defects (off-flavours), and overall evaluation were assessed by the trained panel. Sensory analysis was performed in triplicate.

### 3.6. Statistical Analysis

Significant differences and homogeneous groups of means were established by analysis of variance (ANOVA). When the difference between mean values was significant, a test of comparison of means was performed using the Tukey method (univariate analysis). Mean values and standard deviation are reported. IBM SPSS version 19 for Windows was used in the statistical treatment of the data.

## 4. Conclusions

The use of HHP for the preservation of processed olives is designed as an effective treatment to control the population of microorganisms. HHP treatments of 4 and 6 min were chosen because they are commercially suitable. Treatment times longer than 10 min do not make sense because the cost/benefit ratio would be very low, and could cause damage to the structure of the fruit. Therefore, the conditions chosen are a balance between economic feasibility and the optimisation of the treatment to preserve as many phenolic compounds as possible HHP at 400 MPa for 4 and 6 min does not adversely affect the quality of Spanish-style table olives, although colour and texture decrease significantly during the shelf life. Up to 117 days, the olives presented good quality characteristics to be marked. It should be noted that conventional P treatment negatively affected texture and phenolic composition, compared to HHP. The application of different conditions of pressure/time during HHP treatment did not cause significant differences in the sensory attributes. Furthermore, fermentation with *S. cerevisiae* has positive effects to produce high quality table olives, as well as the addition of OLE, which led to an increase in phenolic compounds in table olives during the storage period.

The results of this experimentation could also be extended to olive varieties different than ‘Carrasqueña’. Presumably, to apply this technology conditions to other olive varieties, time and/or pressure conditions of high hydrostatic pressure should be slightly adapted, due to slightly different texture characteristics of the different olive varieties.

## Figures and Tables

**Table 1:** Results for physical–chemical parameters (mean ± standard deviation). F: spontaneous fermentation of olives; F1: olives fermented with OLE + spontaneous fermentation; F2: olives fermented with OLE + *S. cerevisiae;* P: pasteurization treatment; HHP4: high hydrostatic pressure treatment (400 MPa, 4 min); HHP6: high hydrostatic pressure (400 MPa, 6 min).

Process	Time (Days)	Treatment	pH	Free Acidity (% Lactic Acid)	Total Chlorides (% NaCl)
F	0 (*)	Untreated	3.4 ± 0.0 nsA	0.3 ± 0.0 nsB	4.2 ± 0.1 nsB
		Untreated	3.4 ± 0.0 nsB	0.3 ± 0.0 nsA	4.2 ± 0.1 nsA
		Untreated	3.4 ± 0.0 nsB	0.3 ± 0.0 nsNS	4.2 ± 0.1 nsNS
	117	FP	3.5 ± 0.0 bB	0.3 ± 0.1 nsA	4.6 ± 0.1 bC
		FHHP4	3.3 ± 0.0 aB	0.3 ± 0.1 nsA	4.5 ± 0.2 bB
		FHHP6	3.3 ± 0.0 aB	0.3 ± 0.2 nsNS	4.4 ± 0.1 aNS
	300	FP	3.4 ± 0.0 bB	0.3 ± 0.1 nsB	4.1 ± 0.3 nsA
		FHHP4	3.2 ± 0.0 aA	0.4 ± 0.0 nsB	4.3 ± 0.1 nsA
		FHHP6	3.2 ± 0.0 aA	0.3 ± 0.1 nsNS	4.4 ± 0.1 nsNS
F1	0 (*)	Untreated	3.6 ± 0.5 nsC	0.3 ± 0.3 nsA	4.2 ± 0.4 nsA
		Untreated	3.6 ± 0.5 nsC	0.3 ± 0.3 nsA	4.2 ± 0.4 nsA
		Untreated	3.6 ± 0.5 nsC	0.3 ± 0.3 nsA	4.2 ± 0.4 nsA
	117	F1P	3.5 ± 0.0 bC	0.3 ± 0.2 nsC	4.5 ± 0.1 nsB
		F1HHP4	3.4 ± 0.0 aB	0.3 ± 0.2 nsB	4.4 ± 0.1 nsB
		F1HHP6	3.4 ± 0.0 aB	0.3 ± 0.3 nsB	4.4 ± 0.1 nsC
	300	F1P	3.4 ± 0.0 bB	0.3 ± 0.0 aB	4.2 ± 0.0 aA
		F1HHP4	3.3 ± 0.0 aA	0.3 ± 0.0 aB	4.4 ± 0.1 bB
		F1HHP6	3.3 ± 0.0 aA	0.4 ± 0.0 bC	4.2 ± 0.1 aB
F2	0 (*)	Untreated	3.4 ± 0.4 nsA	0.3 ± 0.0 nsA	4.1± 0.7 nsA
		Untreated	3.4 ± 0.4 nsB	0.3 ± 0.0 nsA	4.1± 0.7 nsA
		Untreated	3.4 ± 0.4 nsB	0.3 ± 0.0 nsA	4.1± 0.7 nsA
	117	F2P	3.5 ± 0.0 bA	0.3 ± 0.2 nsB	4.5 ± 0.1 bB
		F2HHP4	3.3 ± 0.0 aA	0.3 ± 0.2 nsB	4.0 ± 0.2 aA
		F2HHP6	3.4 ± 0.0 bA	0.3 ± 0.1 nsB	4.4 ± 0.2 bB
	300	F2P	3.6 ± 0.0 bB	0.4 ± 0.0 bC	4.7 ± 0.1 bC
		F2HHP4	3.3 ± 0.0 aA	0.4 ± 0.0 aC	4.3 ± 0.1 aB
		F2HHP6	3.3 ± 0.1 aA	0.4 ± 0.0 aC	4.3 ± 0.1 aB

(*) Day 0: Physical–chemical parameters before conservation treatments. Different small letters in the same row indicate statistically significant differences (Tukey’s Test, *p* < 0.05) among conservation treatments of the same day. Different capital letters in the same column indicate statistically significant differences (Tukey’s Test, *p* < 0.05) among different storage time.

**Table 2:** Colour parameters (mean ± standard deviation). F: spontaneous fermentation of olives; F1: olives fermented with OLE + spontaneous fermentation; F2: olives fermented with OLE + *S. cerevisiae;* P: pasteurization treatment; FHHP4: high hydrostatic pressure treatment (400 MPa, 4 min); FHHP6: high hydrostatic pressure (400 MPa, 6 min).

Process	Time (Days)	Treatment	L*	a*	b*
F	0 (*)	Untreated	57.0 ± 2.9 nsB	−6.5 ± 3 nsA	40.5 ± 4.8 nsB
		Untreated	57.0 ± 2.9 nsB	−6.5 ± 3 nsA	40.5 ± 4.8 nsB
		Untreated	57.0 ± 2.9 nsB	−6.5 ± 3 nsNS	40.5 ± 4.8 nsB
	117	FP	56.1 ± 3.2 bB	−5.0 ± 1.5 aAB	39.3 ± 3.9 cB
		FHHP4	50.7 ± 3.6 aA	−1.6 ± 1.4 bC	31.4 ± 4.3 bB
		FHHP6	49.4 ± 3.6 aA	−0.4 ± 2.0 cNS	27.5 ± 5.9 aA
	300	FP	52.0 ± 0.4 nsA	−3.4 ± 1.9 aB	34.9 ± 3.3 bA
		FHHP4	54.8 ± 0.2 nsB	−3.3 ± 2.1 aB	35.3 ± 3.0 bA
		FHHP6	54.4 ± 0.3 nsB	−0.8 ± 2.3 bNS	29.2 ± 3.1 aA
F1	0 (*)	Untreated	61.1 ± 3.1 nsB	−8.6 ± 1.3 nsA	44.1 ± 3.7 nsB
		Untreated	61.1 ± 3.1 nsB	−8.6 ± 1.3 nsA	44.1 ± 3.7 nsB
		Untreated	61.1 ± 3.1 nsB	−8.6 ± 1.3 nsA	44.1 ± 3.7 nsB
	117	F1P	54.3 ± 2.5 bA	−6.7 ± 2.3 aB	37.9 ± 3.0 bA
		F1HHP4	47.9 ± 3.0 aA	−0.8 ± 1.5 bB	29.9 ± 4.0 aA
		F1HHP6	46.8 ± 3.8 aA	−0.6 ± 1.6 bB	28.3 ± 4.9 aA
	300	F1P	56.4 ± 0.6 cA	−5.3 ± 1.6 aB	41.8 ± 2.7 bA
		F1HHP4	44.8 ± 0.3 bA	−0.3 ± 1.2 bB	27.6 ± 4.1 aA
		F1 HHP6	42.5 ± 0.2 aA	−0.4 ± 1.1 bB	28.8 ± 3.5 aA
F2	0 (*)	Untreated	59.2 ± 5.0 nsB	−8.6 ± 1.5 nsNS	42.3 ± 3.8 nsB
		Untreated	59.2 ± 5.0 nsB	−8.6 ± 1.5 nsA	42.3 ± 3.8 nsC
		Untreated	59.2 ± 5.0 nsB	−8.6 ± 1.558 nsA	42.3 ± 3.8 nsB
	117	F2P	56.4 ± 3.3 bB	−7.1 ± 2.0 aNS	40.9 ± 3.6 bB
		F2HHP4	50.7 ± 3.1 aB	−1.8 ± 1.3 bB	33.6 ± 4.4 aB
		F2HHP6	51.6 ± 3.0 aB	−1.5 ± 1.5 bB	34.0 ± 4.1 aA
	300	F2P	45.3 ± 0.2 bA	−7.0 ± 0.5 aNS	29.1 ± 2.1 nsA
		F2HHP4	42.2 ± 0.1 aA	−0.6 ± 1.4 bB	27.3 ± 2.7 nsA
		F2HHP6	45.7 ± 0.5 bA	−0.4 ± 0.1 nsB	30.0 ± 2.7 nsA

(*) Day 0: Colour parameters before the conservation treatments. Different small letters in the same row indicate statistically significant differences (Tukey’s Test, *p* < 0.05) among conservation treatment of the same day. Different capital letters in the same column indicate statistically significant differences (Tukey’s Test, *p* < 0.05) among different storage time.

**Table 3:** Phenolic profile of table olives (mg kg^−1^) during the storage period (mean ± standard deviation). F: spontaneous olive fermentation; F1: olives fermented with OLE + spontaneous fermentation; F2: olives fermented with OLE + *S. cerevisiae;* P: pasteurization treatment; HHP4: high hydrostatic pressure treatment (400 MPa, 4 min); HHP6: high hydrostatic pressure (400 MPa, 6 min).

Process	Time (Days)	Treatment	Hydroxytyrosol	Tyrosol	PB1	Vanillic Acid	Oleuropein	Luteolin	Luteolin-7-*O*-g	Verbascoside	*p*-Coumaric Acid
F	0 (*)	Untreated	876 ± 82 nsC	64 ± 10 nsC	15 ± 2 nsC	15 ± 5 nsC	3 ± 1 ns	11 ± 1 nsB	14 ± 2 nsC	32 ± 32 nsC	20 ± 3 nsC
		Untreated	876 ± 82 nsC	64 ± 10 nsC	15 ± 2 nsC	15 ± 5 nsC	3 ± 1 nsNS	11 ± 1 nsNS	14 ± 2 nsC	32 ± 32 nsC	20 ± 3 nsB
		Untreated	876 ± 82 nsC	64 ± 10 nsB	15 ± 2 nsC	15 ± 5 nsC	3 ± 1 nsNS	11 ± 1 ns	14 ± 2 nsC	32 ± 32 nsC	20 ± 3 nsC
	117	FP	606 ± 22 aB	43 ± 5 aB	8 ± 2 aB	10 ± 5 nsB	n.d.	5 ± 1 aA	8 ± 1 aB	21 ± 12 aB	12 ± 2 aB
		FHHP4	813 ± 21 bB	71 ± 6 bB	13 ± 2 bB	12 ± 3 nsB	3 ± 1 nsNS	10 ± 1 bNS	12 ± 1 bB	31 ± 12 bB	18 ± 3 cB
		FHHP6	805 ± 22 bB	65 ± 5 bB	13 ± 2 bB	12 ± 3 nsB	3 ± 1 nsNS	9 ± 1 b	12 ± 2 bB	26 ± 15 bB	15 ± 2 bB
	300	FP	509 ± 20 aA	33 ± 4 aA	5 ± 1 aA	6 ± 2 nsA	n.d.	n.d.	3 ± 1 aA	11 ± 10 aA	6 ± 1 aA
		FHHP4	753 ± 19 cA	62 ± 5 cA	7 ± 2 bA	6 ± 2 nsA	n.d.	5 ± 1 b	6 ± 1 cA	16 ± 6 cA	13 ± 1 cA
		FHHP6	721 ± 18 bA	55 ± 3 bA	8 ± 1 bA	5 ± 2 nsA	n.d.	4 ± 1 a	5 ± 1 bA	13 ± 10 bA	11 ± 1 bA
F1	0 (*)	Untreated	1801 ± 247 nsC	102 ± 10 nsC	46 ± 1 nsC	33 ± 4 nsC	30 ± 5 nsC	18 ± 1 nsB	49 ± 10 nsC	32 ± 2 nsB	30 ± 5 nsC
		Untreated	1801 ± 247 nsC	102 ± 10 nsC	46 ± 1 nsC	33 ± 4 nsC	30 ± 5 nsC	18 ± 1 nsC	49 ± 10 nsC	32 ± 2 nsC	30 ± 5 nsB
		Untreated	1801 ± 112 nsC	102 ± 10 nsC	46 ± 1 nsC	33 ± 4 nsC	30 ± 5 nsC	18 ± 1 nsC	49 ± 10 nsC	32 ± 2 nsC	30 ± 5 nsB
	117	F1P	1121 ± 20 aB	74 ± 2 aB	20 ± 2 aB	11 ± 1 aB	15 ± 5 aB	7 ± 1 aA	25 ± 2 aB	9 ± 1 aA	12 ± 1 aB
		F1HHP4	1725 ± 12 cB	90 ± 1 bB	40 ± 2 cB	27 ± 2 cB	25 ± 4 bB	15 ± 2 bB	37 ± 3 cB	26 ± 2 bB	29 ± 1 cB
		F1HHP6	1675 ± 11 bB	86 ± 3 bB	30 ± 3 bB	24 ± 2 bB	26 ± 5 bB	15 ± 2 bB	32 ± 2 bB	24 ± 1 bB	25 ± 2 bB
	300	F1P	995 ± 11 aA	55 ± 1 aA	16 ± 1 aA	6 ± 1 aA	10 ± 4 aA	6 ± 1 aA	15 ± 2 aA	7 ± 1 aA	9 ± 1 aA
		F1HHP4	1621 ± 10 bA	75 ± 1 cA	27 ± 2 bA	20 ± 1 bA	15 ± 3 cA	12 ± 1 bA	28 ± 2 bA	19 ± 1 cA	19 ± 1 bA
		F1HHP6	1611 ± 10 bA	60 ± 2 bA	25 ± 1 bA	19 ± 1 bA	13 ± 6 bA	12 ± 1 bA	26 ± 1 bA	16 ± 1 bA	19 ± 1 bA
F2	0 (*)	Untreated	1540 ± 114 nsC	90 ± 10 nsC	35 ± 6 nsB	25 ± 5 nsB	26 ± 3 nsB	14 ± 2 nsB	40 ± 5 nsC	31 ± 38 ns	30 ± 5 nsC
		Untreated	1540 ± 114 nsC	90 ± 10 nsC	35 ± 6 nsC	25 ± 5 nsC	26 ± 3 nsB	14 ± 2 nsNS	40 ± 5 nsC	31 ± 38 ns	30 ± 5 nsC
		Untreated	1540 ± 114 nsC	90 ± 10 nsB	35 ± 6 nsC	25 ± 5 nsC	26 ± 3 nsB	14 ± 2 nsNS	40 ± 5 nsC	31 ± 38 ns	30 ± 5 nsC
	117	F2P	902 ± 21 aB	52 ± 3 aB	12 ± 1 aA	8 ± 1 aA	10 ± 1 aA	6 ± 1 aA	21 ± 2.1 aB	14 ± 1 aB	10 ± 1 aB
		F2HHP4	1452 ± 15 cB	82 ± 2 cB	28 ± 1 bB	22 ± 1 cB	27 ± 1 bB	15 ± 1 cNS	35 ± 2 cB	29 ± 1 cB	28 ± 2 cB
		F2HHP6	1395 ± 12 bB	75 ± 3 bB	26 ± 3 bB	17 ± 1 bB	25 ± 2 bB	13 ± 2 bNS	31 ± 3 bB	25 ± 3 bB	23 ± 2 bB
	300	F2P	811 ± 12 aA	41 ± 2 aA	10 ± 1 aA	6 ± 1 aA	8 ± 1 aA	5 ± 1 aA	16 ± 2 aA	11 ± 1 aA	5 ± 1 aA
		F2HHP4	1312 ± 11 cA	61 ± 1 cA	22 ± 1 bA	17 ± 1 cA	22 ± 1 bA	13 ± 1 bNS	29 ± 2 cA	21 ± 1 bA	21 ± 1 cA
		F2HHP6	1222 ± 11 bA	56 ± 3 bA	19 ± 1 bA	15 ± 1 bA	18 ± 1 bA	12 ± 1 bNS	25 ± 1 bA	18 ± 1 bA	17 ± 1 bA

(*) Day 0: Phenolic profile before conservation. Different small letters in the same row indicate statistically significant differences (Tukey’s Test, *p* < 0.05) among conservation treatment of the same day. Different capital letters in the same column indicate statistically significant differences (Tukey’s Test, *p* < 0.05) among different storage times. PB1: procyanidin B1. n.d.: non detected.

## Data Availability

The authors confirm that the data supporting the findings of this study are available within the article and the raw data that support the findings are available from the corresponding author, upon reasonable request.
